# Effects of vitamin A restriction on carcass characteristics, antioxidant capacity, meat quality and meat storage period of Yanbian yellow cattle

**DOI:** 10.5713/ab.250783

**Published:** 2026-03-11

**Authors:** Xinxin Zhang, CongCong Zhang, Jixuan Song, Jinhui Bai, Beibei Hao, Zewen Wu, Shengxue Sima, Jiahui Zhang, Mengdi Chen, Yue He, Lina Hou, Guangjun Xia

**Affiliations:** 1 Agriculture College, Yanbian University, Yanji, China; 2 Engineering Research Center of North-East Cold Region Beef Cattle Science & Technology Innovation, Ministry of Education, Yanbian University, Yanji, China; 3 Institute of Animal Science, Jilin Academy of Agricultural Science, Changchun, China; 4 Yanbian Korean Autonomous Prefecture Livestock Station, Yanji, China

**Keywords:** Antioxidant Capacity, Beef Quality, Intramuscular Fat Content, Marbling Scores, Restricted Feeding Time

## Abstract

**Objective:**

This study was conducted to determine effects of dietary vitamin A (VA) level and duration on intramuscular fat (IMF), meat quality, storage stability, and antioxidant gene expression in Yanbian yellow cattle.

**Methods:**

Twenty 15-month-old Yanbian yellow cattle (314.13±13.30 kg) were assigned to five treatments: CON (supplemental VA 2,200 IU/kg DM), NVA1 (0 IU/kg DM supplemental VA for 180 d), NVA2 (0 IU/kg DM supplemental VA for 240 d), LVA1 (supplemental VA 1,100 IU/kg DM for 180 d) and LVA2 (supplemental VA 1,100 IU/kg DM for 240 d). Growth performance, carcass traits, physicochemical characteristics, and storage stability were measured. Serum biochemical and muscle antioxidant indexes were analyzed, and the mRNA expression of antioxidant-related genes (*FOXO1*, *GSTA1*, *SOD*) was quantified by quantitative polymerase chain reaction. Statistical significance was set at p<0.05.

**Results:**

All VA-restricted groups showed higher IMF and marbling score, and lower muscle fiber diameter, drip loss, and shear force than the CON group (p<0.05). Serum SOD and glutathione peroxidase levels in the NVA1/NVA2 group were lower than those in the LVA groups and CON (p<0.05). During storage, NVA2/LVA1/LVA2 had lower drip loss and shear force on days 1, 3, and 5 (p<0.05); on day 7, b* was higher in NVA1/NVA2 than CON, and drip loss, cooking loss, and shear force were lower in NVA2, LVA1, and LVA2 than CON (p<0.05). At the transcriptional level, antioxidant-related genes were upregulated across all VA-restricted groups, with *FOXO1* and *GSTA1* peaking in LVA1 and *SOD* elevated in all restricted groups (p<0.05).

**Conclusion:**

Restricting dietary VA to 50% of the recommended level for 180 days significantly improved marbling, tenderness, and oxidative stability without compromising growth performance. These findings highlight a feasible nutritional strategy to enhance beef quality and extend shelf life in Yanbian yellow cattle.

## INTRODUCTION

Marbling, the visible pattern of fat interspersed between muscle fibers in beef, is a significant indicator of premium beef quality. It presents as white specks or streaks created by adipose tissue between muscle fibers [1]. Intramuscular fat (IMF), the material foundation for marbling, is positively correlated with beef color, flavor, and juiciness [2]. Higher IMF content corresponds with elevated marbling scores [3]. Corbin et al [4] found that consumer preference for beef increased as IMF content rose across 10 quality categories and marbling levels.

Vitamin A is a vital, fat-soluble vitamin. Roels [5] initially suggested a connection between vitamin A and fat metabolism, and subsequent studies indicated that vitamin A could influence marbling quantity. Restricted intake of vitamin A has been shown to notably affect IMF content [6]. Kruk et al [7] found that adding low-dose vitamin A supplements to feed can increase the number and size of marble spots, thus promoting the formation of marble patterns and affecting the IMF. Oka et al [8] noticed that the content of vitamin A in feed was negatively correlated with beef marbling. In addition, Gorocica et al [9] found that limiting the vitamin A dosage in the diet did not significantly affect the carcass weight, meat quality or back fat thickness of Angus hybrid offspring, but it did improve the marbling score of beef. In summary, adding a low dose of vitamin A to fodder can increase IMF deposition and marbling scores. However, the optimal supplemental dosage for specific cattle breeds remains undetermined, and the antioxidant function of vitamin A has not been adequately investigated. Although Palacios found that vitamin A has some antioxidant capacity [10], and subsequent research by Daniel et al [11] implyed that vitamin A restriction might be a management strategy to improve beef quality and extend shelf life. Nevertheless, the impact of restricting vitamin A intake in finishing diets on lipid oxidation and color stability of stored meat remains inconclusive. Compared to other local breeds, Yanbian yellow cattle, one of China’s five outstanding local breeds, has exhibited superior meat quality traits, including marbling patterns [12]. However, the meat quality is characterized by instability, which significantly limits the production of high-quality beef from Yanbian yellow cattle. Currently, the optimal supplementation level of vitamin A during the finishing period for Yanbian yellow cattle has not been established. Moreover, there is a lack of research on the role of vitamin A in delaying beef oxidation and preserving meat quality during storage via antioxidant effects, which severely restricts the industrial development of high-grade beef.

Therefore, the objective of this study was to investigate the effects of vitamin A restriction feeding on the production performance, fat deposition, marbling, meat quality antioxidant capacity, and shelf life in Yanbian yellow cattle, and to determine the appropriate level of its addition and time of restriction feeding in the production, as well as the optimal level of vitamin A added to reduce the oxidation of beef. To provide a reference for the application of vitamin A in beef cattle production and sales, it is of great significance to meet consumers’ demand for high-grade and high-quality beef.

## MATERIALS AND METHODS

### Animals and treatments

Twenty healthy and disease-free 15-month-old Yanbian Yellow castrated cattle with similar weights (314.13±13.30 kg) were selected from Muluo Livestock, Longjing City, Yanbian Korean Autonomous Prefecture, Jilin Province. The cattle were randomly divided into five groups: one control (CON group) and four experimental groups, each consisting of four animals. The control group (CON) was supplemented with 2,200 IU/kg DM of VA according to the Nutrient Requirements of Beef Cattle (Nutritional Requirements of Beef Cattle 8th Revision). Treatment group 1 (NVA1) and treatment group 2 (NVA2) were not supplemented VA for 180 days and 240 days, respectively, and then recovered to the same level of VA as the CON group. Treatment group 3 (LVA1) and treatment group 4 (LVA2) were supplemented with 1,100 IU/kg DM of VA for 180 days and 240 days, respectively, and then recovered to the same of VA level as the CON group.

A pretrial period of 15 d was followed by a formal experimental period of 15 months. The cattle were raised to the age of 30 months, and standard commercial slaughter practices were applied. The composition and nutritional levels of the diets are presented in Supplement 1.

### Feeding, management and growth performance

The experimental cattle were provided with free access to water and ad libitum consumption of roughage. A commercial vitamin A premix, purchased from DSM Company, stored in a cool, dry, and dark place.

Feeding was administered twice daily, at 7:00 AM and 3:00 PM. Before each feeding, the premix was mixed with concentrate feed. Fasting body weights (BWs) were recorded at the start and end of the period to calculate average daily gain (ADG).

### Slaughter and carcass determination and sampling

At the end of restriction, jugular blood was collected into sterile tubes, centrifuged (4°C), and serum aliquots were light-protected and stored in liquid nitrogen. After an 18 h fast, cattle were electrically stunned and slaughtered by exsanguination under standard commercial procedures; carcasses were washed, halved, while hide, head, distal limbs, tail, reproductive organs, and surrounding fat were removed. Lean and bone were weighed to calculate dressing yield, lean percentage, and meat:bone ratio. The longissimus dorsi muscle (LD) (12th–13th rib) was sampled. The longissimus muscle area (LMA) was traced on tracing paper and quantified with a transparent grid, and backfat thickness was measured perpendicularly by caliper at the three-quarter point of the LD cross-section near the spine. Marbling score was graded on China’s 5-point scale [13]. LD samples were aseptically collected, and snap-frozen in liquid nitrogen.

### Analysis of meat quality traits

Meat quality traits were evaluated as described by Yu et al [14], with slight adjustments. LDs were aged 48 h at 4°C, then evaluated for pH (pH-STAR; MATTHAUS) and color (L*, a*, b*; OPTO-LAB; MATTHAUS) with instruments calibrated before use. Drip loss was determined on standardized strips (5×3×2 cm) suspended in sealed bags at 4°C for 24 h; cooking loss was calculated after heating to a 75°C–80°C core temperature, cooling, blotting, and reweighing. Shear force was measured with a C-LM3B meat tenderness meter by cutting parallel to fiber orientation. Moisture, crude protein, fat, and ash were analyzed as described by Luan et al [15]. For the 4°C storage trial, pH, color, drip loss, cooking loss, and shear force were recorded on days 1, 3, 5, and 7 using the same procedures. All measurements were performed in triplicate.

### Measurement of muscle fiber diameter

Muscle fiber diameter was determined using a histological method as described by Zhang et al [16], with slight modifications. Briefly, muscle tissues were fixed in a 10% formaldehyde solution for more than 24 h, rinsed in water for 24 h, and then dehydrated in a graded series of alcohol solutions. Next, xylene and anhydrous ethanol were mixed to decolorize (1:1), and then pure xylene was used for secondary decolorization. The treated tissues were embedded in paraffin, sliced, attached to slides, deparaffinized, and subjected to HE staining and neutral gum sealing. Microscopic images were captured for muscle morphology observation, and ImageJ software was used to measure the muscle fiber diameter in beef.

### Biochemical indicators analysis

Serum biochemistry was analyzed by a contract laboratory (Beijing Huaying Biotechnology) using an A6 semi-automatic biochemical analyzer (Beijing Shansheng Technology) and commercial kits (Beijing Huaying Biotechnology). Assays followed manufacturers’ instructions: total protein (TP; HY-50067; biuret, 546 nm), albumin (ALB; HY-50068; bromocresol green, 630 nm), total cholesterol (TC; HY-50061; COD-PAP, 500 nm), triglycerides (TG; HY-50062; GPO-PAP, 500 nm), high-density lipoprotein (HDL; HY-50070; selective detergent method), low-density lipoprotein (LDL; HY-50071; selective detergent method), glucose (GLU; HY-50063; glucose oxidase–peroxidase, 500 nm), urea nitrogen (UREA; HY-N0015; urease/GLDH kinetic, 340 nm), aspartate aminotransferase (AST; HY-50053; rate method with MDH/NADH coupling, 340 nm), and alanine aminotransferase (ALT; HY-50052; rate method with LDH/NADH coupling, 340 nm).

### Antioxidant index analysis

In a service company (Beijing Huaying Biotechnology), commercial kits (Beijing Huaying Biotechnology) and A6 semi-automatic biochemical analyzer (Beijing Shansheng Technology) were used to determine the antioxidant indexes in serum and muscle.

Commercial kits for malondialdehyde (MDA; HY-M0003), glutathione peroxidase (GSH-Px; HY-M0004), catalase (CAT; HY-M0018), superoxide dismutase (SOD; HY-M0001), total antioxidant capacity (T-AOC; HY-60021) (Beijing Huaying Biotechnology) were used with an A6 semi-automatic biochemical analyzer (Beijing Shansheng Technology) according to the manufacturers’ instructions.

### RNA extraction and quantitative polymerase chain reaction analysis

Total RNA was extracted using the Eastep Super Total RNA Extraction Kit provided by Shanghai Promega Company, and the RNA concentration was assessed using an ultraviolet spectrophotometer. First-strand cDNA synthesis was performed using the FastKing cDNA First-Strand Synthesis Kit (TIANGEN). All procedures were performed in accordance with the protocols provided by the respective kits. These procedures were performed as described by Chen et al [17].

Specific primers were designed using Primer Premier 5.0 software based on GenBank-published cattle gene sequences (Supplement 2; Tianjin GENEWIZ Biology Science and Technology, synthesized all primers).The polymerase chain reaction (PCR) conditions were as follows: an initial denaturation at 95°C for 15 min, followed by 40 cycles of denaturation at 95°C for 10 s, annealing at 60°C for 20 s, and extension at 75°C for 25 s. A final extension was performed at 95°C for 15 s, annealing at 55°C for 15 s, and extension at 95°C for 15 s.

The relative gene expression levels were calculated using the 2^−ΔΔCT^ method, where ΔCt target gene = Ct target gene – Ct reference gene, and ΔΔCt = ΔCt experimental group – ΔCt control group. Each sample was run in triplicate.

### Statistical analysis

Graphs were generated using Excel. A one-way analysis of variance was conducted using SPSS 26.0. In the case of significant differences, Duncan’s multiple comparison test was used. The results were presented as mean±standard error. A p-value of less than 0.05 was considered statistically significant.

## RESULTS

### Growth performance and carcass traits

Dietary vitamin A restriction did not significantly affect the growth performance (initial BW, final BW, ADG) (Supplement 3; p>0.05). Similarly, carcass traits, including carcass weight, dressing percentage, net meat percentage, bone weight, meat-bone ratio, weight of high-grade beef parts, and the percentage of high-grade cuts in live weight, were not significantly different from those of the CON group (Supplement 4; p>0.05).

### Fat deposition and marbling score of beef

**Figure 1:**
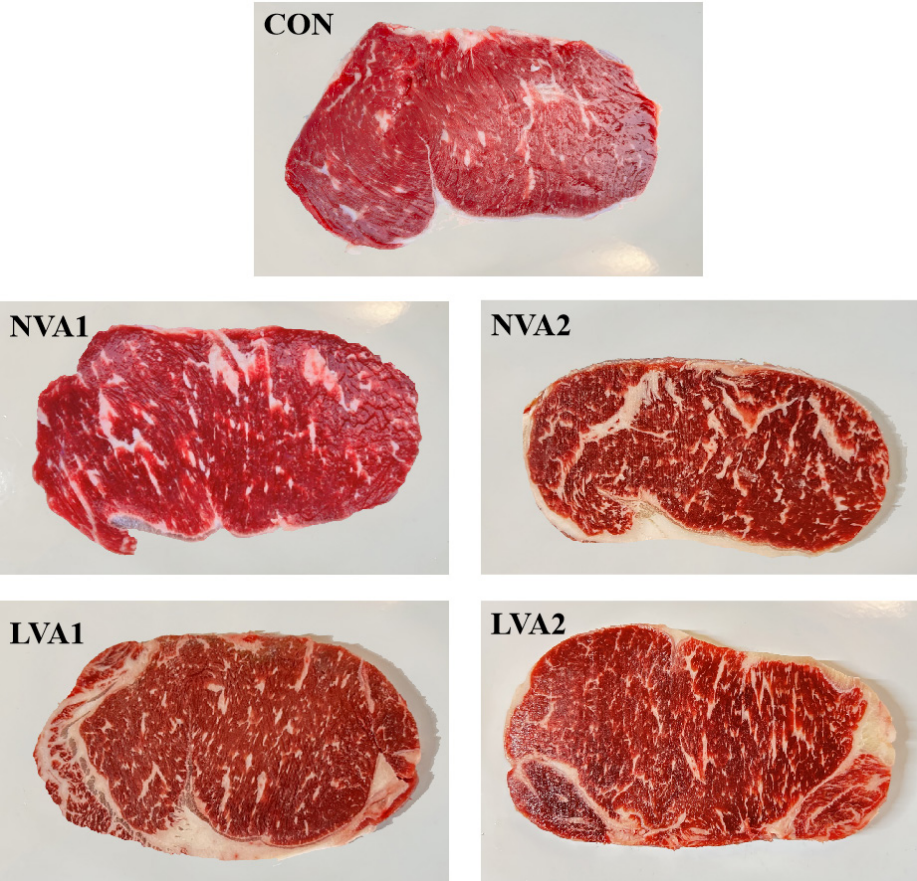
Representative cross-sectional image of the longissimus dorsi muscle at the 12th–13th rib level. CON, supplemental VA 2,200 IU/kg DM; NVA1, supplemental VA 0 IU/kg DM for 180 d; NVA2, supplemental VA 0 IU/kg DM for 240 d; LVA1, supplemental VA 1,100 IU/kg DM for 180 d; LVA2, supplemental VA 1,100 IU/kg DM for 240 d.

Fat deposition and marbling score in Yanbian yellow cattle were shown in Table 1 and Figure 1. Marbling scores in the LVA1 group were significantly higher than those in the CON, NVA1, and NVA2 groups (p<0.05). The marbling score of the LVA2 group was significantly higher than that of the CON group (p<0.05). Compared with the CON group, the marbling scores of the NVA1, NVA2, LVA1, and LVA2 groups increased by 16.67%, 27.67%, 55.67%, and 39.00%, respectively. The IMF contents of the NVA1, NVA2, LVA1, and LVA2 groups were significantly higher than those of the CON group (p<0.05), with increases of 54.21%, 56.99%, 53.93%, and 50.00%, respectively. No significant differences were observed in LMA and backfat thickness among the groups (p>0.05).

### Muscle fiber diameter

As shown in Table 2 and Supplement 5, muscle fiber diameters were significantly smaller in all treatment groups (NVA1, NVA2, LVA1, LVA2) compared to the CON group (p<0.05), indicating that vitamin A restriction effectively reduced muscle fiber size.

### Physicochemical characteristics

The effects of vitamin A on the physicochemical characteristics of Yanbian Yellow beef were shown in Table 3. The drip loss rates of the NVA2, LVA1, and LVA2 groups were significantly lower than that of the CON group (p<0.05). Additionally, drip loss rate in the LVA1 group was significantly lower than that in the NVA1 group (p<0.05). The shear forces of the NVA2, LVA1, and LVA2 groups were significantly lower than that of the CON group (p<0.05), and both the LVA1 and LVA2 groups exhibit significantly lower shear forces than that of the NVA1 group (p<0.05). However, no significant differences were observed among the groups in terms of pH, meat color, cooking loss rates, moisture, protein, or ash contents (p>0.05).

### Serum biochemical and antioxidant indexes

In Table 4, restricting vitamin A levels in the daily diet did not significantly affect the serum biochemical indexes of Yanbian yellow cattle (p*>*0.05). There was no significant difference (p>0.05) in SOD concentration between the groups of LVA1 and LVA2 compared with the CON group, but the concentration of SOD in NVA1 and NVA2 groups was significantly lower than that of the CON group (p<0.05), and that in NVA2 were significantly lower than that of LVA1 group (p<0.05). In addition, the concentration of GSH-PX in NVA1 and NVA2 was significantly lower than that in CON, LVA1 and LVA2 groups (p<0.05).

### Muscle antioxidant indexes

The effect of vitamin A on muscle antioxidant indexes of Yanbian yellow cattle was shown in Supplement 6. Compared with the CON group, T-AOC, CAT, SOD, GSH-PX and MDA content of the four experimental groups were not significantly different (p>0.05).

### Antioxidant gene expression profile in longissimus dorsi muscle

**Figure 2:**
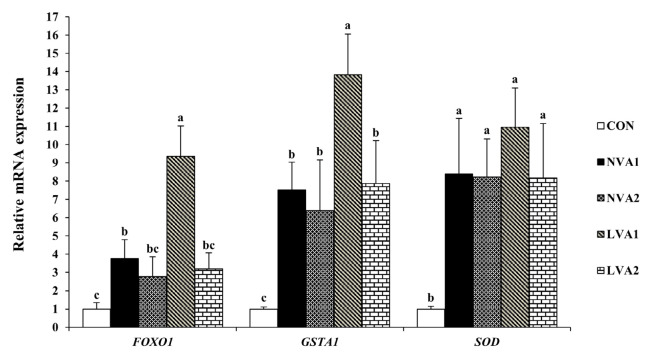
Effect of vitamin A on the relative mRNA expression of *FOXO1*, *GSTA1* and *SOD* genes in the longissimus dorsi muscle of Yanbian yellow cattle. CON, supplemental VA 2,200 IU/kg DM; NVA1, supplemental VA 0 IU/kg DM for 180 d; NVA2, supplemental VA 0 IU/kg DM for 240 d; LVA1, supplemental VA 1,100 IU/kg DM for 180 d; LVA2, supplemental VA 1,100 IU/kg DM for 240 d. ^a–c^ Different superscript letters within a row represent significant differences (p<0.05).

As shown in Figure 2, relative mRNA expression of *FOXO1*, *GSTA1*, and *SOD* in the LD were upregulated in all experimental groups compared with the CON group. For the *FOXO1* gene, the relative mRNA expression level in the LVA1 group was significantly higher than that in the CON, NVA1, NVA2, and LVA2 groups (p<0.05). Moreover, the NVA1 group exhibited a significantly higher *FOXO1* expression compared to the CON group (p<0.05). For the *GSTA1* gene, the mRNA expression in the LVA1 group was significantly higher than that in the NVA1, NVA2, LVA2, and CON groups (p<0.05). The NVA1, NVA2, and LVA2 groups all demonstrated significantly higher *GSTA1* expression than the CON group. As for the *SOD* gene, all experimental groups had significantly higher expression levels compared with the CON group. These findings further confirm that vitamin A restriction enhances the intrinsic antioxidant defense system in the muscle of Yanbian yellow cattle, which is beneficial for maintaining meat quality stability during storage.

### Changes in meat quality characteristics during storage

As shown in Supplement 7, the pH value decreased initially and then increased over storage. All groups exhibited similar trends, with no significant differences detected among them (p>0.05).

**Figure 3:**
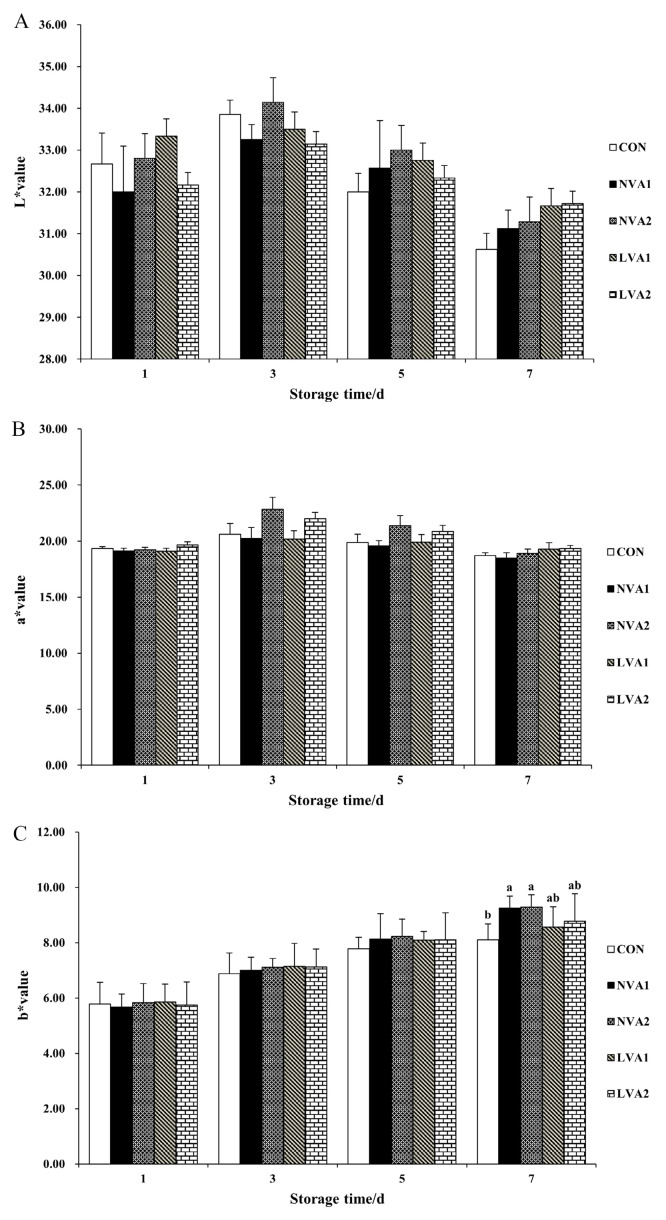
Effect of vitamin A on meat color of Yanbian yellow beef during storage. (A) L* (lightness) value, (B) a* (redness) value, (C) b* (yellowness) value of meat color. CON, supplemental VA 2,200 IU/kg DM; NVA1, supplemental VA 0 IU/kg DM for 180 d; NVA2, supplemental VA 0 IU/kg DM for 240 d; LVA1, supplemental VA 1,100 IU/kg DM for 180 d; LVA2, supplemental VA 1,100 IU/kg DM for 240 d. ^a,b^ Different superscript letters within a row represent significant differences (p<0.05).

The evolution of meat color (L*, a*, b*) was shown in Figures 3A–3C. The L* and a* values were not significantly affected by storage time (p>0.05). In contrast, the b* value increased progressively, with the NVA1 and NVA2 groups exhibiting significantly higher values than the CON group by day 7 (p<0.05).

**Figure 4:**
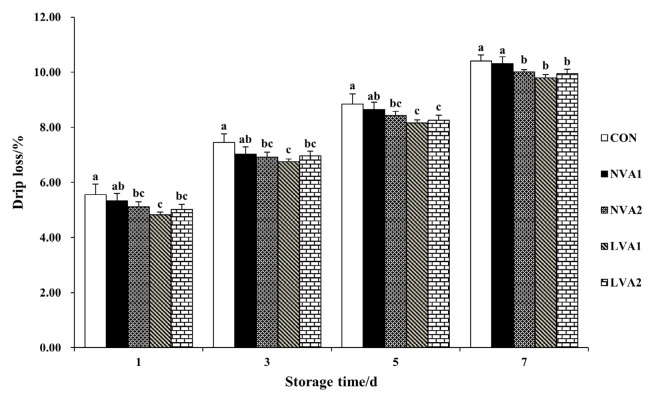
Effect of vitamin A on drip loss of Yanbian yellow beef during storage. CON, supplemental VA 2,200 IU/kg DM; NVA1, supplemental VA 0 IU/kg DM for 180 d; NVA2, supplemental VA 0 IU/kg DM for 240 d; LVA1, supplemental VA 1,100 IU/kg DM for 180 d; LVA2, supplemental VA 1,100 IU/kg DM for 240 d. ^a–c^ Different superscript letters within a row represent significant differences (p<0.05).

Vitamin A restriction effectively reduced drip loss in Figure 4. The NVA2, LVA1, and LVA2 groups were lower than CON group on days 1, 3, and 5 (p<0.05), and on day 7 these three groups were lower than both CON and NVA1 groups (p<0.05), with the LVA1 group showing the lowest value.

**Figure 5:**
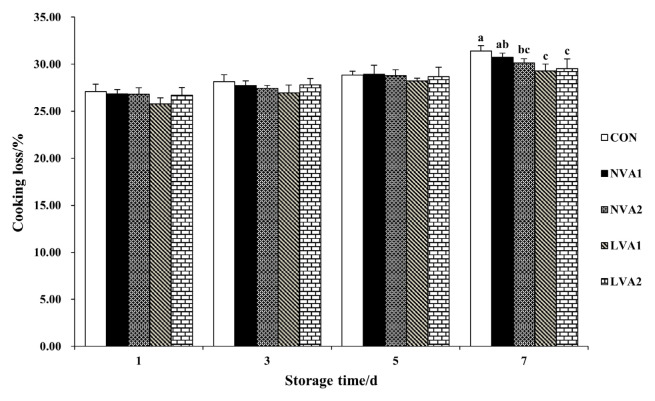
Effect of vitamin A on cooking loss of Yanbian yellow beef during storage. CON, supplemental VA 2,200 IU/kg DM; NVA1, supplemental VA 0 IU/kg DM for 180 d; NVA2, supplemental VA 0 IU/kg DM for 240 d; LVA1, supplemental VA 1,100 IU/kg DM for 180 d; LVA2, supplemental VA 1,100 IU/kg DM for 240 d. ^a–c^ Different superscript letters within a row represent significant differences (p<0.05).

According to Figure 5, it was learned that restricted feeding of vitamin A was able to reduce the cooking loss rate of muscle. Compared with the CON group, the NVA2 group, LVA1 group and LVA2 group decreased significantly on the 7th day of storage (p<0.05).

**Figure 6:**
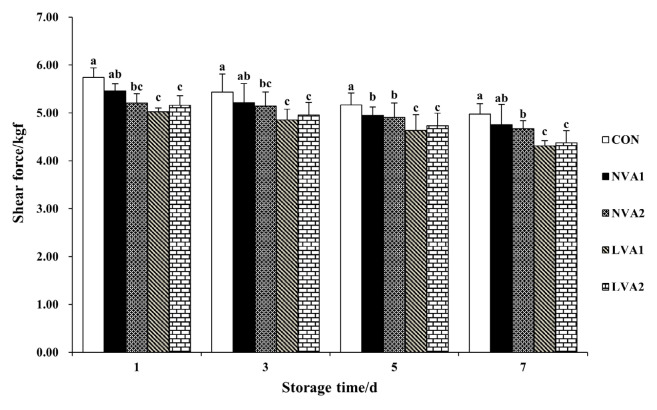
Effect of vitamin A on shear force of Yanbian yellow beef during storage. CON, supplemental VA 2,200 IU/kg DM; NVA1, supplemental VA 0 IU/kg DM for 180 d; NVA2, supplemental VA 0 IU/kg DM for 240 d; LVA1, supplemental VA 1,100 IU/kg DM for 180 d; LVA2, supplemental VA 1,100 IU/kg DM for 240 d. ^a–c^ Different superscript letters within a row represent significant differences (p<0.05).

As shown in Figure 6, shear force exhibited a general decline during storage. On days 1 and 3, the LVA1, LVA2, and NVA2 groups had significantly lower shear force than the CON group, with the LVA1 and LVA2 groups also being significantly lower than the NVA1 group (p<0.05). By day 5, all treatment groups showed significantly lower values than CON, and the LVA groups were significantly lower than the NVA groups (p<0.05). This trend continued on day 7, with the LVA groups being significantly lower than all other groups, and the NVA2 group lower than CON (p<0.05). The LVA1 group consistently demonstrated the lowest shear force throughout the storage period.

## DISCUSSION

Regarding beef production, previous studies consistently indicate that restricting vitamin A intake does not significantly affect the growth performance [18–20] or carcass traits [21,22] of steers. Similarly, this study found no significant differences in growth performance, carcass traits, or serum biochemical parameters. This suggests that the VA restriction regimen in this trial had a limited impact on overall metabolic homeostasis, with its effects more likely to be manifested in meat quality-related traits. Therefore, the subsequent experimental analysis were conducted.

Marbling score is a critical indicator for assessing beef quality, and the restricted vitamin A strategy plays a pivotal role in the development of marbling and IMF in beef. Wan [23] observed that reducing dietary vitamin A content significantly increased IMF in Limousin-crossbred steers without affecting fat deposition in other regions. On one hand, higher IMF content is generally associated with improved tenderness. Previous studies have found that vitamin A restriction can reduce beef shear force [22], which aligns with the findings of the present study. On the other hand, tenderness is closely related to muscle fiber diameter: smaller fiber diameters typically correspond to lower shear force and show a negative correlation with IMF [24]. Furthermore, increased IMF is believed to help reduce moisture exudation to some extent, thereby decreasing drip loss and imparting meat juiciness [25]. It had to be emphasized that the LVA1 group had the lowest shear force and drip loss, but the best marbling score.

Vitamin A is involved in regulating redox homeostasis in the body and can help scavenge free radicals [26]. After slaughter, endogenous antioxidants are gradually depleted; once insufficient, ROS-driven damage accelerates and impairs meat quality [27]. Although there was no significant difference in muscle antioxidant level in the results of this experiment, the activities of SOD and GSH-Px in serum were significantly reduced in the group without VA. As SOD and GSH-Px are crucial for clearing reactive oxygen species and protecting against oxidative damage [28]. Our results suggest that low-dose vitamin A restriction has a limited effect on antioxidant capacity, while no vitamin A supplementation may weaken antioxidant defense.

Previous research has reported nonlinear responses of antioxidant parameters to vitamin A in poultry [29]. In our study, we observed a non-linear relationship between dietary vitamin A levels and the expression of antioxidant-related genes. Compared with the CON group, the expression of muscle *SOD* was significantly up-regulated in all vitamin A-restricted groups. Meanwhile, the expression levels of *FOXO1* and *GSTA1* peaked in the LVA1 group, while those in the NVA1/NVA2 and LVA2 groups, although relatively lower, still remained higher than the levels observed in the CON group. These results point to changes in the antioxidant network at the transcriptional level, although this does not necessarily mean an immediate improvement in overall antioxidant function. At moderate vitamin A levels, retinoic acid signaling appears to regulate pathways such as Nrf2-ARE and FOXO, which aligns with the increased expression of *SOD* and *GSTA1* observed here [30]. The FOXO family, important transcription factors in oxidative stress response, helps maintain basic antioxidant defense [31]. SOD primarily mediates the superoxide anions, while GSTA1, as a phase II detoxification enzyme, clears lipid peroxides. Under the guidance of FOXO1 and Nrf2 signaling, they form a coordinated antioxidant network [32]. An *in vitro* study using bovine AD-MSCs suggests that exogenous antioxidant treatment can improve redox status while modulating differentiation-related signaling pathways, thereby promoting adipogenic differentiation [33]. Previous studies have also indicated that vitamin A can promote the transformation of bovine skeletal muscle toward an oxidative muscle fiber phenotype, suggesting its potential to enhance oxidative metabolic capacity [34]. Therefore, these transcriptional changes provide a molecular mechanistic explanation for the improved meat stability observed during storage. As the antioxidant system helps mitigate post-slaughter lipid oxidation damage [35], the significant upregulation of antioxidant genes in the LVA1 group enables it to maintain superior quality throughout storage.

Concurrently, the low-dose vitamin A groups (LVA1 and LVA2) demonstrated the most favorable meat quality attributes during storage, characterized by the highest water retention capacity and optimal tenderness. Notably, in this study, the difference became more pronounced in the later stage of storage. By day 7 of storage, the low-dose vitamin A group exhibited significantly lower drip loss and cooking loss rates, along with a significantly lower shear force, compared to the CON group, a result consistent with previous reports [36]. Given that water loss rate and cooking loss rate are closely related to meat tenderness and juiciness [37], this suggests that low-dose vitamin A restriction may enhance meat quality stability during the later stages of storage. The underlying mechanisms appear to be related to IMF content and alterations in muscle microstructure. Existing research suggests that meat with higher IMF content is generally associated with lower drip loss and improved sensory attributes [38]. During storage, myofibrillar restructuring and protein degradation can influence water distribution in muscle and, consequently, water-holding capacity [39]. In this study, vitamin A restriction significantly reduced cooking loss in Yanbian yellow cattle, with the LVA1 group showing the greatest improvement. This further supports the idea that low-level restriction provides a more stable quality advantage, especially in the later stages of storage.

## CONCLUSION

In summary, vitamin A restriction during the finishing period may improve beef quality through two complementary pathways. First, it promote the formation of smaller-diameter muscle fibers, which can help enhance water-holding capacity and tenderness. Second, low-dose restriction induce the upregulation of antioxidant-related genes, potentially extending shelf life. These findings provide useful insight for optimizing vitamin A nutrition strategies during the finishing stage.

## Figures and Tables

**Table 1:** Effects of vitamin A on fat deposition and marbling score of beef in Yanbian yellow cattle

Item	Group^1)^					SEM	p-value

	CON	NVA1	NVA2	LVA1	LVA2		
Marbling score	3.00^c^	3.50^bc^	3.83^b^	4.67^a^	4.17^ab^	0.180	0.010
Intramuscular fat (%)	14.74^b^	22.73^a^	23.14^a^	22.69^a^	22.11^a^	0.851	<0.001
LMA (cm^2^)	115.00	112.33	111.67	114.33	115.67	0.821	0.301
Backfat thickness (cm)	0.91	0.88	0.89	0.89	0.91	0.10	0.924

1) CON, supplemental VA 2,200 IU/kg DM; NVA1, supplemental VA 0 IU/kg DM for 180 d; NVA2, supplemental VA 0 IU/kg DM for 240 d; LVA1, supplemental VA 1,100 IU/kg DM for 180 d; LVA2, supplemental VA 1,100 IU/kg DM for 240 d.

a–c Different superscript letters within a row represent significant differences (p<0.05).

SEM, standard error of the means; LMA, longissimus muscle area.

**Table 2:** Effect of vitamin A on muscle fiber diameter in Yanbian yellow cattle

Item	Group^1)^					SEM	p-value

	CON	NVA1	NVA2	LVA1	LVA2		
Muscle fiber diameter (μm)	52.79^a^	49.06^b^	48.96^b^	49.62^b^	50.07^b^	0.491	0.049

1) CON, supplemental VA 2,200 IU/kg DM; NVA1, supplemental VA 0 IU/kg DM for 180 d; NVA2, supplemental VA 0 IU/kg DM for 240 d; LVA1, supplemental VA 1,100 IU/kg DM for 180 d; LVA2, supplemental VA 1,100 IU/kg DM for 240 d.

a,b Different superscript letters within a row represent significant differences (p<0.05).

SEM, standard error of the means.

**Table 3:** Effects of vitamin A on physicochemical characteristics of Yanbian yellow cattle

Item		Group^1)^					SEM	p-value

		CON	NVA1	NVA2	LVA1	LVA2		
pH		5.53	5.52	5.49	5.50	5.48	0.011	0.451
Meat color^2)^	L*	32.67	32.00	32.80	31.56	32.17	0.659	0.976
	a*	19.33	19.13	19.22	19.09	19.63	0.107	0.566
	b*	5.71	5.67	5.78	5.83	5.67	0.225	0.999
Drip loss (%)		5.56^a^	5.33^ab^	5.12^bc^	4.82^c^	5.02^bc^	0.064	<0.001
Cooking loss (%)		27.08	26.84	26.79	25.79	26.70	0.402	0.911
Shear force (kgf)		5.63^a^	5.45^ab^	5.29^bc^	5.04^c^	5.08^c^	0.053	0.001
Chemical components								
Moisture (%)		65.24	65.67	65.77	66.19	66.56	0.531	0.949
Protein (% of DM)		53.62	55.46	55.18	56.05	57.07	0.674	0.515
Ash (% of DM)		2.76	2.66	2.61	2.51	2.58	0.064	0.770

1) CON, supplemental VA 2,200 IU/kg DM; NVA1, supplemental VA 0 IU/kg DM for 180 d; NVA2, supplemental VA 0 IU/kg DM for 240 d; LVA1, supplemental VA 1,100 IU/kg DM for 180 d; LVA2, supplemental VA 1,100 IU/kg DM for 240 d.

2) L*, lightness; a*, redness; b*, yellowness.

a–c Different superscript letters within a row represent significant differences (p<0.05).

SEM, standard error of the means.

**Table 4:** Effects of vitamin A on serum biochemical and antioxidant indexes of Yanbian yellow cattle

Item	Group^1)^					SEM	p-value

	CON	NVA1	NVA2	LVA1	LVA2		
TP (g/L)	69.83	69.83	74.40	72.38	70.69	0.994	0.574
ALB (g/L)	31.81	31.39	29.18	31.99	29.53	0.666	0.592
TC (mmol/L)	2.31	2.56	2.49	2.62	2.58	0.077	0.840
TG (mmol/L)	0.30	0.39	0.30	0.40	0.38	0.017	0.113
HDL (mmol/L)	1.27	1.32	1.33	1.36	1.35	0.037	0.973
LDL (mmol/L)	0.59	0.59	0.57	0.56	0.56	0.031	0.997
GLU (mmol/L)	4.26	4.28	4.24	4.39	4.54	0.093	0.886
UREA (mmol/L)	3.81	4.16	4.01	4.03	3.80	0.056	0.167
AST (U/L)	89.86	89.20	89.14	89.73	89.90	0.195	0.657
ALT (U/L)	27.52	24.65	22.96	26.53	26.35	1.435	0.934
T-AOC (U/mL)	7.54	6.83	6.92	7.33	7.18	0.098	0.277
CAT (U/mL)	23.65	23.39	23.29	23.52	23.73	0.061	0.120
SOD (U/mL)	76.24^a^	72.08^bc^	70.60^c^	75.20^ab^	74.12^abc^	0.635	0.015
GSH-PX (U/mL)	330.68^a^	286.74^b^	281.29^b^	324.33^a^	318.21^a^	4.810	<0.001
MDA (nmol/mL)	4.29	4.47	4.52	4.59	4.59	0.118	0.971

1) CON, supplemental VA 2,200 IU/kg DM; NVA1, supplemental VA 0 IU/kg DM for 180 d; NVA2, supplemental VA 0 IU/kg DM for 240 d; LVA1, supplemental VA 1,100 IU/kg DM for 180 d; LVA2, supplemental VA 1,100 IU/kg DM for 240 d.

a–c Different superscript letters within a row represent significant differences (p<0.05).

SEM, standard error of the means; TP, total protein; ALB, albumin; TC, total cholesterol; TG, triacylglycerol; HDL, high-density lipoprotein; LDL, low-density lipoprotein; GLU, glucose; UREA, urea nitrogen; AST, aspartate aminotransferase; ALT, alanine aminotransferase; T-AOC, total antioxidant capacity; CAT, catalase; SOD, superoxide dismutase; GSH-Px, glutathione peroxidase; MDA, malondialdehyde.

## Data Availability

Upon reasonable request, the datasets of this study can be available from the corresponding author.

## SUPPLEMENTARY MATERIAL

**Supplement 1**. Concentrate composition and nutrient level (dry matter basis).

**Supplement 2**. Primer sequences.

**Supplement 3**. Effects of vitamin A on growth performance in Yanbian Yellow Cattle.

**Supplement 4**. Effects of vitamin A on carcass traits of Yanbian Yellow Cattle.

**Supplement 5.** Sectional diagram of muscle fiber tissue morphology (10×).

**Supplement 6**. Effects of vitamin A on antioxidant indexes in the muscle of Yanbian Yellow Cattle.

**Supplement 7**. Effect of vitamin A on pH value of Yanbian Yellow Beef during storage.

## References

[1] Harper GS Pethick DW The physiology of marbling: what is it, and why does it develop Marbling Symposium: Proceedings of a CRC Conference 2001 Oct 9–10 Coffs Harbour, Australia Cooperative Research Centre for Cattle and Beef Quality 2001 36 45

[2] Wang Y Wang Q Dai C Effects of dietary energy on growth performance, carcass characteristics, serum biochemical index, and meat quality of female Hu lambs Anim Nutr 2020 6 499 506 10.1016/j.aninu.2020.05.008 33364466 PMC7750792

[3] McBee JL Wiles JA Influence of marbling and carcass grade on the physical and chemical characteristics of beef J Anim Sci 1967 26 701 4 10.2527/jas1967.264701x

[4] Corbin CH O’Quinn TG Garmyn AJ Sensory evaluation of tender beef strip loin steaks of varying marbling levels and quality treatments Meat Sci 2015 100 24 31 10.1016/j.meatsci.2014.09.009 25299587

[5] Roels OA The fifth decade of vitamin A research Am J Clin Nutr 1969 22 903 7 10.1093/ajcn/22.7.903 5797058

[6] Gregoire FM Smas CM Sul HS Understanding adipocyte differentiation Physiol Rev 1998 78 783 809 10.1152/physrev.1998.78.3.783 9674695

[7] Kruk ZA Bottema MJ Reyes-Veliz L Forder REA Pitchford WS Bottema CDK Vitamin A and marbling attributes: intramuscular fat hyperplasia effects in cattle Meat Sci 2018 137 139 46 10.1016/j.meatsci.2017.11.024 29182958

[8] Oka A Maruo Y Miki T Yamasaki T Saito T Influence of vitamin A on the quality of beef from the Tajima strain of Japanese Black cattle Meat Sci 1998 48 159 67 10.1016/S0309-1740(97)00086-7 22062888

[9] Gorocica-Buenfil MA Fluharty FL Bohn T Schwartz SJ Loerch SC Effect of low vitamin A diets with high-moisture or dry corn on marbling and adipose tissue fatty acid composition of beef steers J Anim Sci 2007 85 3355 66 10.2527/jas.2007-0172 17709781

[10] Palacios A Piergiacomi VA Catalá A Vitamin A supplementation inhibits chemiluminescence and lipid peroxidation in isolated rat liver microsomes and mitochondria Mol Cell Biochem 1996 154 77 82 10.1007/BF00248464 8717420

[11] Daniel MJ Dikeman ME Arnett AM Hunt MC Effects of dietary vitamin A restriction during finishing on color display life, lipid oxidation, and sensory traits of longissimus and triceps brachii steaks from early and traditionally weaned steers Meat Sci 2009 81 15 21 10.1016/j.meatsci.2008.07.003 22063957

[12] Zhang X Xu H Zhang C Effects of vitamin A on Yanbian yellow cattle and their preadipocytes by activating AKT/mTOR signaling pathway and intestinal microflora Animals 2022 12 1477 10.3390/ani12121477 35739812 PMC9219514

[13] Zhang X Ge F Gao H Analysis of growth, slaughter performance and meat quality of Yiling cattle Shandong Agric Sci 2023 55 163 70

[14] Yu X Li C Zhao Z Zhang Y Effects of slaughter age on the quality of gannan yak meat: analysis of edible quality, nutritional value, and GC × GC-ToF-MS of the longissimus dorsi muscle Food Sci Nutr 2025 13 e70381 10.1002/fsn3.70381 40521073 PMC12163752

[15] Luan J Jin Y Zhang T Effects of dietary vitamin E supplementation on growth performance, slaughter performance, antioxidant capacity and meat quality characteristics of finishing bulls Meat Sci 2023 206 109322 10.1016/j.meatsci.2023.109322 37666007

[16] Zhang R Xu M Xu R Identification of biomarkers for meat quality in Sichuan goats through 4D label-free quantitative proteomics Animals 2025 15 887 10.3390/ani15060887 40150416 PMC11939516

[17] Chen M Zhang C Wu Z Bta-miR-365-3p-targeted FK506-binding protein 5 participates in the AMPK/mTOR signaling pathway in the regulation of preadipocyte differentiation in cattle Anim Biosci 2024 37 1156 67 10.5713/ab.23.0328 38665092 PMC11222839

[18] Arnett AM Dikeman ME Daniel MJ Olson KC Jaeger J Perrett J Effects of vitamin A supplementation and weaning age on serum and liver retinol concentrations, carcass traits, and lipid composition in market beef cattle Meat Sci 2009 81 596 606 10.1016/j.meatsci.2008.10.017 20416585

[19] Gorocica-Buenfil MA Fluharty FL Reynolds CK Loerch SC Effect of dietary vitamin A concentration and roasted soybean inclusion on marbling, adipose cellularity, and fatty acid composition of beef J Anim Sci 2007 85 2230 42 10.2527/jas.2006-780 17468427

[20] Gorocica-Buenfil MA Fluharty FL Reynolds CK Loerch SC Effect of dietary vitamin A restriction on marbling and conjugated linoleic acid content in Holstein steers J Anim Sci 2007 85 2243 55 10.2527/jas.2006-781 17468420

[21] Kruk ZA Bottema CDK Davis JJ Effects of vitamin A on growth performance and carcass quality in steers Livest Sci 2008 119 12 21 10.1016/j.livsci.2008.02.008

[22] Marti S Realini CE Bach A Pérez-Juan M Devant M Effect of vitamin A restriction on performance and meat quality in finishing Holstein bulls and steers Meat Sci 2011 89 412 8 10.1016/j.meatsci.2011.05.003 21641120

[23] Wan FC Effects of vitamin A on beef quality of Limousin × Luxi crossbred steers and action mechanisms [dissertation] Chinese Academy of Agricultural Sciences 2005

[24] Fang C Study on the differences of meat quality, muscle fiber and enzyme activity of Longlin Yellow cattle, Holstein cattle and Xilin buffalo [master’s thesis] Guangxi University 2018

[25] Adeyemi KD Sabow AB Shittu RM Karim R Karsani SA Sazili AQ Impact of chill storage on antioxidant status, lipid and protein oxidation, color, drip loss and fatty acids of semimembranosus muscle in goats CyTA J Food 2015 14 405 14 10.1080/19476337.2015.1114974

[26] Mason SA Trewin AJ Parker L Wadley GD Antioxidant supplements and endurance exercise: current evidence and mechanistic insights Redox Biol 2020 35 101471 10.1016/j.redox.2020.101471 32127289 PMC7284926

[27] Zou B Shao L Yu Q Zhao Y Li X Dai R Changes of mitochondrial lipid molecules, structure, cytochrome C and ROS of beef longissimus lumborum and psoas major during postmortem storage and their potential associations with beef quality Meat Sci 2023 195 109013 10.1016/j.meatsci.2022.109013 36334513

[28] Chen R Shao H Lin S Zhang JJ Xu KQ Treatment with Astragalus membranaceus produces antioxidative effects and attenuates intestinal mucosa injury induced by intestinal ischemia-reperfusion in rats Am J Chin Med 2011 39 879 87 10.1142/S0192415X11009275 21905279

[29] Liang JR Dai H Yang HM Yang Z Wang ZY The effect of dietary vitamin A supplementation in maternal and its offspring on the early growth performance, liver vitamin A content, and antioxidant index of goslings Poult Sci 2019 98 6849 56 10.3382/ps/pez432 31350994 PMC8913995

[30] Shi HY Yan SM Guo YM Zhang BQ Guo XY Shi BL Vitamin A pretreatment protects NO-induced bovine mammary epithelial cells from oxidative stress by modulating Nrf2 and NF-κB signaling pathways J Anim Sci 2018 96 1305 16 10.1093/jas/sky037 29669072 PMC6140872

[31] Storz P Forkhead homeobox type O transcription factors in the responses to oxidative stress Antioxid Redox Signal 2011 14 593 605 10.1089/ars.2010.3405 20618067 PMC3038124

[32] Klotz LO Sánchez-Ramos C Prieto-Arroyo I Urbánek P Steinbrenner H Monsalve M Redox regulation of FoxO transcription factors Redox Biol 2015 6 51 72 10.1016/j.redox.2015.06.019 26184557 PMC4511623

[33] Naseem S Xuan MF Hua H Vitamin C and N-acetylcysteine promote bovine adipose-derived mesenchymal stem cell proliferation and differentiation via Akt/mTOR/P70S6K signalling pathway for cultured meat production Anim Biosci 2025 38 2250 65 10.5713/ab.24.0776 40400197 PMC12415370

[34] Wang B Nie W Fu X Neonatal vitamin A injection promotes cattle muscle growth and increases oxidative muscle fibers J Anim Sci Biotechnol 2018 9 82 10.1186/s40104-018-0296-3 30459947 PMC6236944

[35] Faustman C Sun Q Mancini R Suman SP Myoglobin and lipid oxidation interactions: mechanistic bases and control Meat Sci 2010 86 86 94 10.1016/j.meatsci.2010.04.025 20554121

[36] Li J Zou L Wang L Zhao H Wang D Sun P Effects of vitamin A on beef quality of Angus × Qinchuan crossbred steers China Anim Husb Vet Med 2012 39 73 8

[37] Muchenje V Dzama K Chimonyo M Strydom PE Hugo A Raats JG Some biochemical aspects pertaining to beef eating quality and consumer health: a review Food Chem 2009 112 279 89 10.1016/j.foodchem.2008.05.103

[38] Tao Y Ma L Li D Tian Y Liu J Liu D Proteomics analysis to investigate the effect of oxidized protein on meat color and water holding capacity in Tan mutton under low temperature storage LWT 2021 146 111429 10.1016/j.lwt.2021.111429

[39] Apple JK Kegley EB Galloway DL Wistuba TJ Rakes LK Duration of restraint and isolation stress as a model to study the dark-cutting condition in cattle J Anim Sci 2005 83 1202 14 10.2527/2005.8351202x 15827265

