# Supplementary material for: Effects of vitamin A restriction on carcass characteristics, antioxidant capacity, meat quality and meat storage period of Yanbian yellow cattle
Source: Anim Biosci. 2026 Mar 11;39(6):250783. doi: 10.5713/ab.250783 (PMC13243974; doi:10.5713/ab.250783)
Supplement: Supplementary file 1 [file ab-250783-Supplementary-1.pdf]

Supplement 1. Concentrate composition and nutrient level (dry matter basis).

Ingredient composition	Content, %	Nutrient levels	Content, %
Corn	64.00	Crude Protein	20.70
Soybean Meal	19.20	Ether Extract	4.46
Wheat Bran	10.00	Crude Ash	4.07
Sodium Bicarbonate	1.00	Neutral Detergent Fiber	18.43
Sodium Chloride	0.80	Acid Detergent Fiber	12.06
Permix[1]	5.00	Calcium	0.74
Total	100.00	Phosphorus	0.57
		NEg[2](MJ/kg)	4.37

Notes:[1] Premix is provided per kg of concentrate: Vitamin D_3 80000IU, Vitamin E 200mg, Vitamin K 10mg, Biotin 2mg, Fe 2000mg, Mn 2000mg, Zn 2000mg, Cu 400mg, I 16mg, Se 100mg, Co 20mg, Ethoxyquin 500mg;

[2] NEg (net energy for growth) was estimated from the measured values of the dietary ingredients [based on the Ministry of Agriculture of P.R. China (2018)].
