# Supplementary material for: Effects of vitamin A restriction on carcass characteristics, antioxidant capacity, meat quality and meat storage period of Yanbian yellow cattle
Source: Anim Biosci. 2026 Mar 11;39(6):250783. doi: 10.5713/ab.250783 (PMC13243974; doi:10.5713/ab.250783)
Supplement: Supplementary file 2 [file ab-250783-Supplementary-2.pdf]

Supplement 2. Primer sequences.

Genes	GenBank ID	Sequence (5'-3')	Amplicon size (bp)
FOXO1	NM_001206083.1	F: GCAGATTTACGAGTGGATGG<br>R: TTGAATTCTTCCAGCCCG	86
GSTA1	NM_001078149.1	F: TTCCCTCTGCTAAAGGCCCT<br>R: CTTCCTCTGGCTGCCAGG	84
SOD	NM_174615.2	F: CAAAGGGAGATACAGTCGTGGTAAC<br>R: TTTGGACAGAGGATTAAAGTGAGGAC	137
β-actin	NM_173979.3	F: AGGCATCCTGACCCTCAAGTA<br>R: GCTCGTTGTAGAAGGTGTGGT	95

Note: F: Forward primer; R: Reverse primer
