# Supplementary material for: Effects of vitamin A restriction on carcass characteristics, antioxidant capacity, meat quality and meat storage period of Yanbian yellow cattle
Source: Anim Biosci. 2026 Mar 11;39(6):250783. doi: 10.5713/ab.250783 (PMC13243974; doi:10.5713/ab.250783)
Supplement: Supplementary file 3 [file ab-250783-Supplementary-3.pdf]

Supplement 3. Effects of vitamin A on growth performance in Yanbian Yellow Cattle.

Item[1]	Group[2]					SEM[3]	p-value
	CON	NVA1	NVA2	LVA1	LVA2		
Initial BW, kg	316.73	305.97	313.20	311.07	323.70	3.556	0.660
Final BW, kg	701.07	681.70	684.43	693.77	703.57	6.038	0.466
ADG, kg/d	0.85	0.83	0.82	0.85	0.84	0.008	0.315

Notes: [1] IBW=initial body weight; FBW=final body weight; ADG=average daily gain.

[2] CON, supplemental VA 2200 IU/kg DM; NVA1, supplemental VA 0 IU/kg DM for 180 d; NVA2, supplemental VA 0 IU/kg DM for 240 d; LVA1, supplemental VA 1100 IU/kg DM for 180 d; LVA2, supplemental VA 1100 IU/kg DM for 240 d.

[3] SEM, standard error of the means.
