# Supplementary material for: Effects of vitamin A restriction on carcass characteristics, antioxidant capacity, meat quality and meat storage period of Yanbian yellow cattle
Source: Anim Biosci. 2026 Mar 11;39(6):250783. doi: 10.5713/ab.250783 (PMC13243974; doi:10.5713/ab.250783)
Supplement: Supplementary file 4 [file ab-250783-Supplementary-4.pdf]

Supplement 4. Effects of vitamin A on carcass traits of Yanbian Yellow Cattle.

Item	Group[1]					SEM[2]	p-value
	CON	NVA1	NVA2	LVA1	LVA2		
Carcass weight, kg	403.14	389.76	390.76	398.73	405.73	9.439	0.727
Dressing Percentage, %	57.86	57.16	57.09	57.47	57.31	0.377	0.981
Net meat percentage, %	48.83	48.36	48.36	48.75	48.95	0.398	0.745
Bone weight, kg	60.93	59.97	59.77	60.50	61.20	0.409	0.827
Meat-bone ratio	5.62	5.50	5.54	5.59	5.63	0.051	0.950
Weight of high-grade beef parts							
Ribeye, kg	18.93	18.12	18.30	18.60	19.85	0.375	0.680
High rib, kg	20.01	18.41	18.78	19.51	20.06	0.478	0.808
Tenderloin, kg	4.93	4.70	4.84	4.96	5.02	0.211	0.994
Striploin, kg	13.36	13.11	12.79	13.36	13.88	0.398	0.958
Percentage of high-grade cuts in live weight, %	8.16	8.04	8.11	8.14	8.36	0.146	0.980

Notes: [1] CON, supplemental VA 2200 IU/kg DM; NVA1, supplemental VA 0 IU/kg DM for 180 d; NVA2, supplemental VA 0 IU/kg DM for 240 d; LVA1, supplemental VA 1100 IU/kg DM for 180 d; LVA2, supplemental VA 1100 IU/kg DM for 240 d.

[2] SEM, standard error of the means.
