# Supplementary material for: Effects of vitamin A restriction on carcass characteristics, antioxidant capacity, meat quality and meat storage period of Yanbian yellow cattle
Source: Anim Biosci. 2026 Mar 11;39(6):250783. doi: 10.5713/ab.250783 (PMC13243974; doi:10.5713/ab.250783)
Supplement: Supplementary file 5 [file ab-250783-Supplementary-5.pdf]

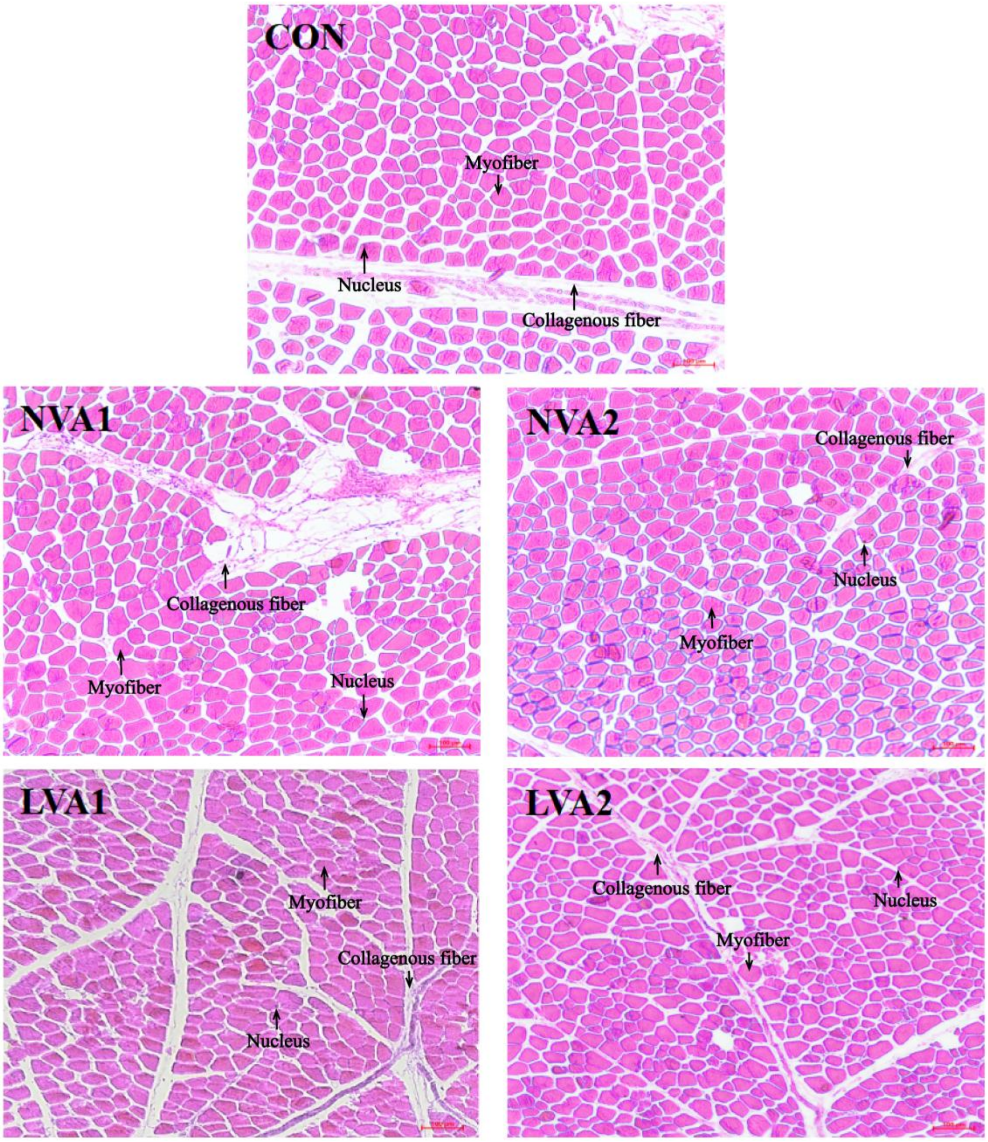

Supplement 5. Sectional diagram of muscle fiber tissue morphology (10×). Scale bar = 200 μm. CON, supplemental VA 2200 IU/kg DM; NVA1, supplemental VA 0 IU/kg DM for 180 d; NVA2, supplemental VA 0 IU/kg DM for 240 d; LVA1, supplemental VA 1100 IU/kg DM for 180 d; LVA2, supplemental VA 1100 IU/kg DM for 240 d.
