# Supplementary material for: Effects of vitamin A restriction on carcass characteristics, antioxidant capacity, meat quality and meat storage period of Yanbian yellow cattle
Source: Anim Biosci. 2026 Mar 11;39(6):250783. doi: 10.5713/ab.250783 (PMC13243974; doi:10.5713/ab.250783)
Supplement: Supplementary file 6 [file ab-250783-Supplementary-6.pdf]

Supplement 6. Effects of vitamin A on antioxidant indexes in the muscle of Yanbian Yellow Cattle.

Item[1]	Group[2]					SEM[3]	p-value
	CON	NVA1	NVA2	LVA1	LVA2		
T-AOC, U/mL	5.12	5.06	5.06	5.12	5.37	0.105	0.921
CAT, U/mL	27.21	27.11	27.37	27.48	27.43	0.272	0.996
SOD, U/mL	69.19	71.20	71.64	70.86	72.28	0.418	0.176
GSH-PX, U/mL	192.82	190.76	192.13	192.66	193.60	0.568	0.662
MDA, nmol/mL	8.54	8.61	8.47	8.48	8.39	0.073	0.931

Notes: [1] T-AOC, total antioxidant capacity; CAT, catalase; SOD, superoxide dismutase; GSH-Px, glutathione peroxidase; MDA, malondialdehyde.

[2] CON, supplemental VA 2200 IU/kg DM; NVA1, supplemental VA 0 IU/kg DM for 180 d; NVA2, supplemental VA 0 IU/kg DM for 240 d; LVA1, supplemental VA 1100 IU/kg DM for 180 d; LVA2, supplemental VA 1100 IU/kg DM for 240 d.

[3] SEM, standard error of the means.
