# Supplementary material for: Effects of vitamin A restriction on carcass characteristics, antioxidant capacity, meat quality and meat storage period of Yanbian yellow cattle
Source: Anim Biosci. 2026 Mar 11;39(6):250783. doi: 10.5713/ab.250783 (PMC13243974; doi:10.5713/ab.250783)
Supplement: Supplementary file 7 [file ab-250783-Supplementary-7.pdf]

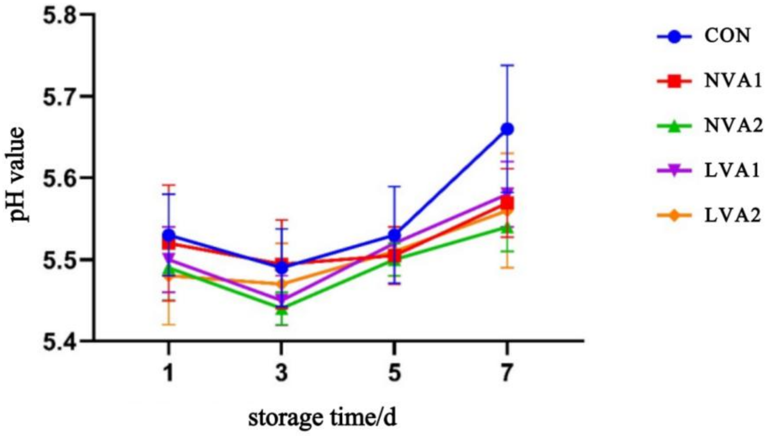

Supplement 7. Effect of vitamin A on pH value of Yanbian Yellow Beef during storage. The legend is on the right, representing the pH value of CON group, NVA1 group, NVA2 group, LVA1 group and LVA2 group on the 1.3.5.7 day. CON, supplemental VA 2200 IU/kg DM; NVA1, supplemental VA 0 IU/kg DM for 180 d; NVA2, supplemental VA 0 IU/kg DM for 240 d; LVA1, supplemental VA 1100 IU/kg DM for 180 d; LVA2, supplemental VA 1100 IU/kg DM for 240 d.
